# CT-based AI model for predicting therapeutic outcomes in ureteral stones after single extracorporeal shock wave lithotripsy through a cohort study

**DOI:** 10.1097/JS9.0000000000001820

**Published:** 2024-06-17

**Authors:** Huancheng Yang, Xiang Wu, Weihao Liu, Zhong Yang, Tianyu Wang, Weifan You, Baiwei Ye, Bingni Wu, Kai Wu, Haoyang Zeng, Hanlin Liu

**Affiliations:** a Department of Radiology, The Third Affiliated Hospital of Shenzhen University (Luohu Hospital Group); b Shantou University Medical College, Shantou University, Shantou; c Shenzhen University Medical College, Shenzhen University, Shenzhen; d Department of Radiology, Shenzhen People's Hospital, Shenzhen, People’s Republic of China

**Keywords:** deep learning, extracorporeal shock wave lithotripsy, machine learning, ureteral stones

## Abstract

**Objectives:**

Exploring the efficacy of an artificial intelligence (AI) model derived from the analysis of computed tomography (CT) images to precisely forecast the therapeutic outcomes of singular-session extracorporeal shock wave lithotripsy (ESWL) in the management of ureteral stones.

**Methods:**

A total of 317 patients diagnosed clinically with ureteral stones were included in this investigation. Unenhanced CT was administered to the participants within the initial fortnight preceding the inaugural ESWL. The internal cohort consisted of 250 individuals from a local healthcare facility, whereas the external cohort comprised 67 participants from another local medical institution. The proposed framework comprises three main components: an automated semantic segmentation model developed using 3D U-Net, a feature extractor that integrates radiomics and autoencoder techniques, and an ESWL efficacy prediction model trained with various machine learning algorithms. All participants underwent thorough postoperative follow-up examinations 4 weeks hence. The efficacy of ESWL was defined by the absence of stones or residual fragments measuring ≤2 mm in KUB X-ray assessments. Model stability and generalizability were judiciously validated through a fivefold cross-validation approach and a multicenter external test strategy. Moreover, Shapley Additive Explanations (SHAP) values for individual features were computed to elucidate the nuanced contributions of each feature to the model’s decision-making process.

**Results:**

The semantic segmentation model the authors constructed exhibited an average Dice coefficient of 0.88±0.08 on the external testing set. ESWL classifiers built using Support Vector Machine (SVM), Random Forest (RF), XGBoost (XB), and CatBoost (CB) achieved AUROC values of 0.78, 0.84, 0.85, and 0.90, respectively, on the internal validation set. For the external testing set, SVM, RF, XB, and CB predicted ESWL with AUROC values of 0.68, 0.79, 0.80, and 0.83, respectively, with the last one being the optimal algorithm. The radiomics features and auto-encoder features made significant contributions to the decision-making process of the classification model.

**Conclusions:**

This investigation unmistakably underscores the remarkable predictive prowess exhibited by a scrupulously crafted AI model using CT images to precisely anticipate the therapeutic results of a singular session of ESWL for ureteral stones.

## Background

Highlights

- Integration of advanced imaging analysis: Our study innovatively integrates advanced imaging analysis techniques, including semantic segmentation using 3D U-Net and feature extraction with radiomics and autoencoder methods. This comprehensive approach allows for a detailed analysis of computed tomography images, providing valuable insights into the characteristics of ureteral stones and their response to treatment.
- Multicenter validation strategy: Unlike many previous studies limited to single-center data, we employed a multicenter validation strategy to enhance the robustness and generalizability of our findings. By including participants from multiple medical institutions, we ensure that our AI model’s predictive performance is validated across diverse patient populations and clinical settings.
- Evaluation of machine learning algorithms: Our research evaluates the efficacy of various machine learning algorithms, including Support Vector Machine (SVM), Random Forest (RF), XGBoost (XB), and CatBoost (CB), for predicting therapeutic outcomes in ureteral stone management. This comprehensive analysis allows us to identify CatBoost as the optimal algorithm, highlighting its superior performance in predicting the success of extracorporeal shock wave lithotripsy.
- Interpretability of model decisions: To enhance the interpretability of our AI model, we computed Shapley Additive Explanations (SHAP) values for individual features, elucidating the nuanced contributions of each feature to the model’s decision-making process. This innovative approach provides clinicians with valuable insights into the factors influencing treatment outcomes, facilitating informed decision-making in clinical practice.

Ureteral stones, commonly referred to as urolithiasis, possess the potential to manifest at diverse anatomical loci within the urinary system, encompassing the renal calyces, renal pelvis, and the urethra^1^. This pervasive urological ailment has garnered heightened attention in recent years, a trajectory mirroring the advancements in societal living standards^2^. The clinical tableau of ureteric calculi is delineated by symptoms such as dolor and hematuria, concomitant with complications like fluid accumulation in the ureter and renal pelvis. These nuanced manifestations significantly impinge upon the holistic quality of life for afflicted individuals^3^. As a widely employed modality in the management of ureteral stones^4^, extracorporeal shock wave lithotripsy (ESWL) boasts advantages in terms of convenience, minimal invasiveness, diminished patient discomfort, and expeditious postoperative recovery. Nevertheless, clinical observations underscore that the applicability of ESWL treatment may not be universal for all instances of ureteral stones. Owing to the relatively modest efficacy in stone expulsion associated with ESWL, a substantial cohort of patients necessitates subsequent surgical interventions, such as ureteroscopy and stone fragmentation, subsequent to unsuccessful ESWL procedures. This not only protracts the realization of efficacious treatment but also potentially exacerbates obstruction, heightens the peril of assorted complications, and intensifies patient distress.

Currently, artificial intelligence (AI) models have been fashioned through the utilization of computed tomography (CT) images associated with ureteral stones. Nevertheless, a considerable portion of these models relies upon the manual quantification of radiological manifestations and markers performed by specialists in radiology^5–7^. This procedure, while essential, is both time-intensive and laborious. Despite certain investigations incorporating models for the extraction of distinctive features, they remain contingent upon the meticulous, manual layer-by-layer annotation of the region of interest (ROI). This approach is afflicted by a dearth of automation, susceptibility to label noise, and a pronounced influence of subjectivity, rendering it unsuitable for deployment in clinical workspaces. Additionally, extant models are burdened by the opaqueness of their ‘black-box’ nature^8^, engendering a deficiency in interpretability during the decision-making juncture. This dearth of interpretability undermines trust among clinical practitioners, posing a significant impediment to the broad acceptance and integration of such AI models into clinical practice^9^. Consequently, a preoperative interpretable automated prognosis of the efficacy of ESWL assumes paramount clinical significance.

To the best of our knowledge, there is currently no reliable, fully automated AI model for predicting the therapeutic outcomes of single-session ESWL for ureteral stones. In a bid to rectify these constraints, this study endeavors to scrutinize whether an AI model cultivated from CT images can furnish precise and efficacious intelligent evaluations of the therapeutic effects associated with singular-session ESWL for ureteral stones. The potency of this model resides in its capacity to proffer streamlined and dependable decision support for clinical practice, thereby augmenting patient treatment outcomes.

## Methods

### Patients and treatment

**Figure 1:**
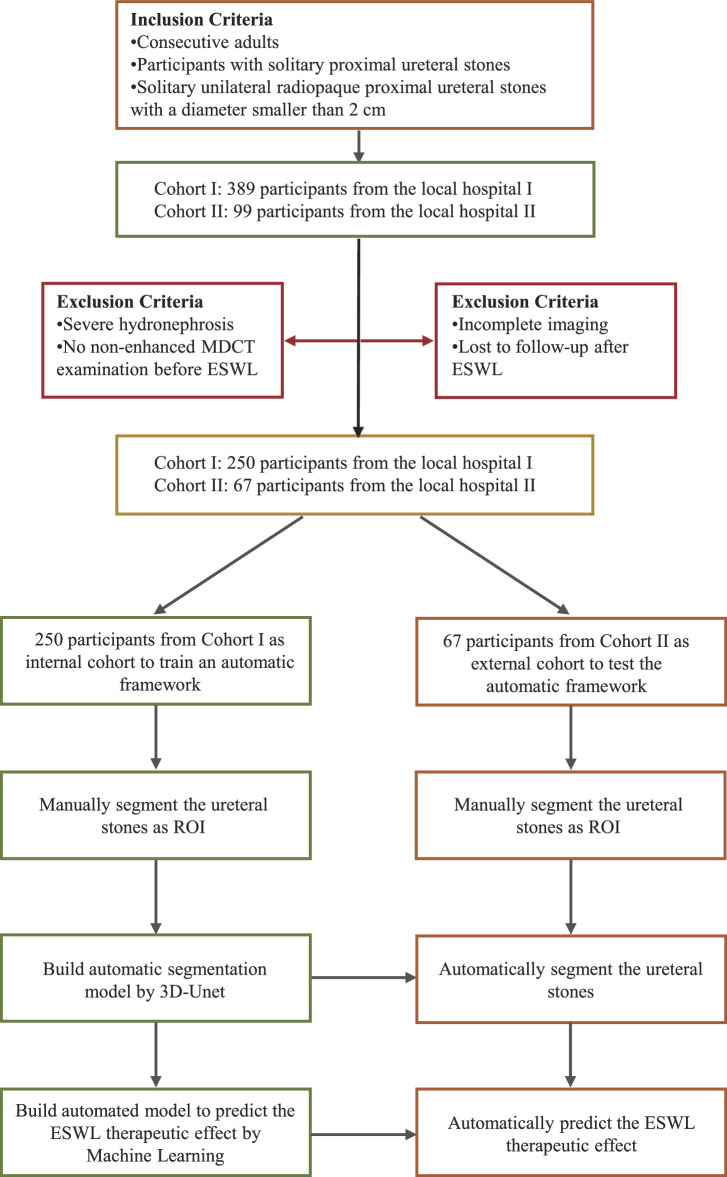
Workflow schema. Showing the inclusion and exclusion criteria for patient selection for in two different local hospitals.

**Figure 2:**
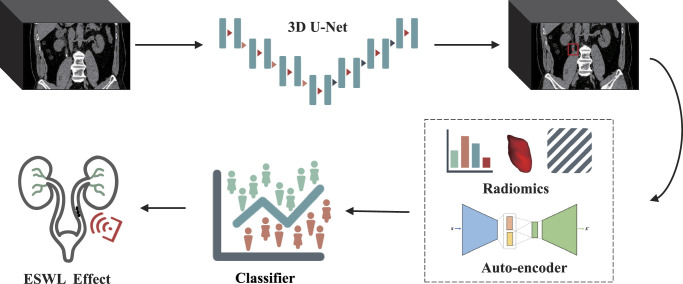
Overall analysis framework.

This study collected a cohort of 488 patients with ureteral stones between October 2023 and March 2024. The stone sizes ranged from 0.5 to 2.0 cm, and all patients underwent non-contrast MDCT scans prior to treatment. The interval from CT scanning to lithotripsy for all patients did not surpass the duration of 2 weeks, and they were subjected to singular-session ESWL intervention. Rigorous criteria for the selection of specimens, as elucidated in Figure 1, were adhered to for the purposes of both inclusion and exclusion. The inclusion criteria were as follows: (1) consecutive adults; (2) participants with solitary ureteral stones in the proximal ureter; (3) solitary unilateral radiopaque proximal ureteral stones with a diameter smaller than 2 cm. Exclusion criteria: (1) severe hydronephrosis; (2) no pre-ESWL nonenhanced MDCT examination; (3) incomplete imaging; (4) lost to follow-up after ESWL. Ultimately, we included a total of 317 patient samples, categorized into an internal cohort (Center 1, Shenzhen People’s Hospital, *n*=250) and an external cohort (Center 2, The Third Affiliated Hospital of Shenzhen University, *n*=67), based on the source. This study strictly adheres to the STROCSS standards^10^ and has received approval from the hospital's Ethics Committee (2023-KYLL-57). The confidential information pertaining to all participants was rendered anonymous to safeguard their privacy. A schematic representation of the AI model is delineated in Figure 2.

### Follow-up and assessment

Four weeks subsequent to ESWL intervention, a postoperative patient follow-up transpired, wherein the assessment of stone clearance predominantly manifested through KUB X-ray imaging. Patients garnered the designation of stone-free (SF) if discerned devoid of residual stone fragments surpassing a diameter of 2 mm. Residual fragments contained within this dimensional ambit were denominated as clinically insignificant residual fragments (CIRFs). In instances where identified residual fragments exhibited a diameter surpassing the stipulated 2 mm threshold, the ESWL treatment was categorically deemed ineffective. In order to ensure uniformity in the diagnostic scrutiny of images, all KUB X-ray representations were subjected to assessment by an identical, seasoned radiologist. If KUB X-ray imaging cannot conclusively confirm the presence or absence of stone fragments, we rely on assessment by two experienced radiologists and urologists, who evaluate the efficacy of ESWL based on several factors: 1. Relief of obstructive symptoms (such as relief of flank pain or assessment of obstruction via renal ultrasound); 2. Presence of visible stone passage in urine; 3. In cases of uncertainty regarding 1 and 2, consideration of follow-up MDCT for comparison.

### Segmentation model for ureteral stones

The entirety of patients underwent MDCT examinations pertaining to the urinary system antecedent to ESWL, and the temporal span spanning from CT scrutiny to lithotripsy did not surpass a fortnight. The data were transcribed into Digital Imaging and Communications in Medicine (DICOM) files in Hounsfield Units (HU) and transformed from DICOM structure to Neuroimaging Informatics Technology Initiative (NIFTI) files deploying the dcm2niix apparatus. A fledgling radiologist meticulously demarcated the peripheries of the ROI. To guarantee the precision of manual delineations, a seasoned radiologist scrutinized all ROIs. Following the acquisition of three-dimensional imagery and segmentation outcomes, we employed the sophisticated 3D U-Net framework in conjunction with a rigorous five-fold cross-validation strategy to meticulously craft a semantic segmentation model tailored for the precise identification and automated delineation of ureteral stones.

To substantiate the precision and accuracy of said segmentation model, we systematically gauged the concurrence between the prognosticated and authentic segmentations through the computation of the Dice coefficient. This coefficient, situated on a continuum from 0 to 1, serves as a prevalent benchmark for the appraisal of automated segmentation model efficacy in tasks of such nature. A heightened Dice coefficient signifies an augmented resemblance between the model’s segmentation outputs and manually annotated results, thereby manifesting superior segmentation performance. The mathematical expression for computing the Dice coefficient is articulated as follows:


Dice coefficient=(2*|A∩B|)/(|A|+|B|)

In this investigation, A signifies the meticulously annotated image sequences within the external cohort, whereas B denotes the image sequences annotated through the automated segmentation model. The Dice coefficient adeptly gauges the resemblance between the segmentation outcomes produced by the model and the manual annotations.

### Feature extraction and model building

This study orchestrated the extraction and evaluation of radiomic attributes, utilizing the open-source computational tool PyRadiomics (v3.0.1) to construct a radiomic feature extractor. A total of 100 radiomic attributes, encompassing texture intricacies, morphological characteristics, and first-order statistical properties, were discerned from the regions containing ureteral stones in each CT image. Additionally, we employed an unsupervised deep learning dimensionality reduction algorithm, the 3D auto-encoder, to construct a 3D auto-encoder feature extractor, which yielded a total of 256 auto-encoder features. Finally, we integrated 100 radiomics features with 256 auto-encoder features, resulting in a total of 356 image features, which were utilized as the comprehensive input features for training the downstream classifiers.

In pursuit of identifying the optimal performing model, we employed a variety of machine learning algorithms, including Support Vector Machine (SVM), Random Forest (RF), XGBoost (XB), and CatBoost (CB), to train a classification model for predicting the efficacy of ESWL (success vs. failure). Furthermore, we utilized ROC testing (bootstrap method) to assess the statistical comparisons between SVM, RF, and XB against CB.

Lastly, in order to evaluate the performance of models trained with input from auto-encoder features, radiomic features, and their combined features, we developed three classification models and conducted a comparative analysis of their performance.

### Interpretability and statistical analysis of the model

To evaluate the contribution of each input feature to the predictive model, this study employed SHAP values to scrutinize the interpretability of the tree model. SHAP, employing a game-theoretic paradigm, facilitates elucidating the intricate mechanisms by which sophisticated machine learning models formulate predictions and delineates the individual contributions of each feature to these predictions. The SHAP values for the extracted features, in the context of predicting the therapeutic outcomes of single-session ESWL for ureteral stones (success vs. failure), were computed and subsequently portrayed in a bar chart, showcasing the top 20 features characterized by the highest SHAP values. This visualization serves to expound upon the influence of each feature on the predictive model. Augmented SHAP values signify an augmented influence of the feature on the model’s decision-making process. Furthermore, the color coding in the bee swarm plot denotes high (red) or low (blue) feature values, with positive SHAP values indicative of a heightened likelihood of the metric being a pivotal predictive factor.

All statistical analyses and experimental procedures in this study were executed utilizing Python (v3.8) and R (v3.6.3). Metric data conforming to a normal distribution are articulated as X±S, whereas non-normally distributed data are delineated by the median (interquartile range). The Dice coefficient was deployed to appraise the concurrence between manually annotated and model-annotated images. For nonquantitative assessments, such as accuracy and AUROC, 95% CIs were employed for precise quantification. Statistical significance was ascribed when the *P*-value was <0.05. Significance was denoted by * if *P*<0.05.

## Results

### Basic characteristics of the participants

This investigation encompassed a collective of 317 subjects, comprising 259 male and 58 female. They were stratified into an intrinsic cohort (*n*=250) and an extrinsic cohort (*n*=67). Within the intrinsic cohort, there were 208 male and 42 female, with an mean age of 48.42±15.33 years. In the extrinsic cohort, there were 51 male and 16 female, with an average age of 51.51±13.13 years. In the internal cohort, 164 patients underwent successful ESWL treatment, while 96 patients experienced treatment failure. In the external cohort, 45 patients had successful ESWL treatments, while 22 patients faced treatment failure. The basic information and CT parameters of the samples are summarized in Table 1.

**Table 1:** Basic characteristics and CT parameters of participant.

Characteristics	Internal cohort	External cohort
Participants	250	67
Age (years)	48.42±15.33*	51.51±13.13*
Sex		
Male	208 (83.20%)	51 (76.12%)
Female	42 (16.80%)	16 (23.88%)
ESWL therapeutic outcomes		
Success	164 (65.60%)	45 (67.16%)
Failure	96 (34.40%)	22 (32.84%)
CT parameters		
Tube voltage (kV)	120	120
Tube current (mA)	240	220
Matrix	512* 512	512* 512
Slice thickness (mm)	0.625	0.625
Pitch	0.92	0.88

* The data with is the mean value±SD.

### Performance of the ESWL treatment outcome prediction model

**Figure 3:**
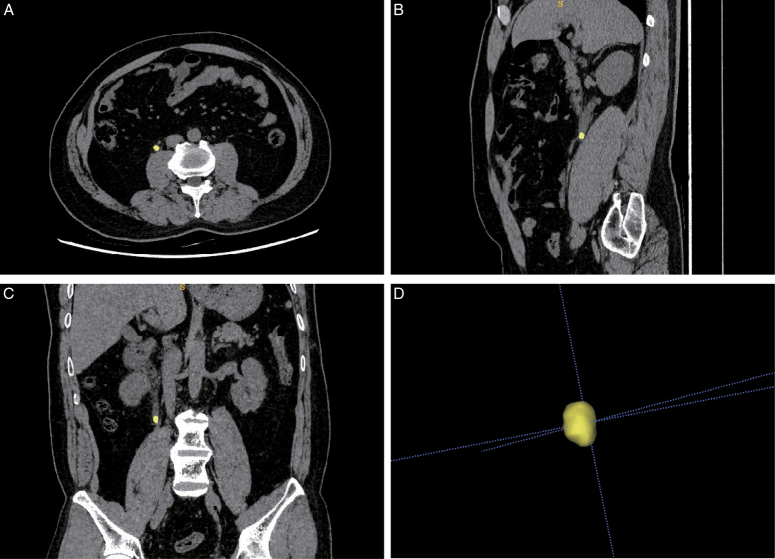
Predictive segmentation in the external test set. In the segmentation results, the yellow color represents the ureteral stones. (A) Axial segmented image. (B) Sagittal segmented image. (C) Coronal segmented image. (D) 3D segmented image.

The semantic segmentation model trained with 3D U-Net adeptly discerns and delineates the three-dimensional domains of urinary stones from CT images. The model attained a Dice coefficient of 0.88±0.08 on the external testing set. Illustrated in Figure 3 is the visual portrayal of the segmentation outcomes for a urinary stone patient from the testing set by this segmentation model, wherein the yellow area signifies the stones identified and segmented.

**Figure 4:**
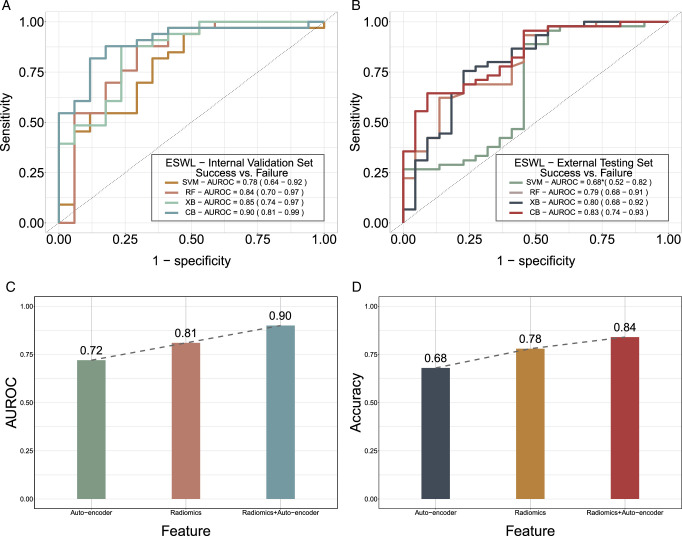
Predictive performances of diverse machine learning algorithms in the internal and external cohorts (A–D). ROC test (bootstrap method) was used for statistical comparison (with * marked if *P*<0.05) between Support Vector Machine (SVM), Random Forest (RF), and XGBoost (XB) algorithms, respectively, with CatBoost (CB) algorithm. Plots (A, B) show the ROC curves of SVM, RF, XB and CB algorithm, in the classification tasks of ESWL (success vs. failure), respectively, in the internal validation set and external testing set. The area under curve and accuracy metrics are presented for different feature extraction solution with the ESWL (success vs. failure) classifications, highlighting the optimal model.

As portrayed in Figure 4A, the ESWL classifiers fashioned utilizing SVM, RF, XB, and CB achieved AUROC values of 0.78, 0.84, 0.85, and 0.90, correspondingly, on the internal validation set. In regard to the external testing set (Fig. 4B), the AUROC values for SVM, RF, XB, and CB were 0.68, 0.79, 0.80, and 0.83, respectively, with CB emerging as the optimal algorithm. As depicted in Figure 4C-D, the optimal ESWL classifiers trained with auto-encoder features, radiomic features, and their amalgamation yielded AUROC values of 0.72±0.1, 0.81±0.1, and 0.90±0.1, sequentially, within the 95% CI, accompanied by accuracies of 0.68, 0.78, and 0.84, respectively.

### The relationship between radiomic features and model decisions

**Figure 5:**
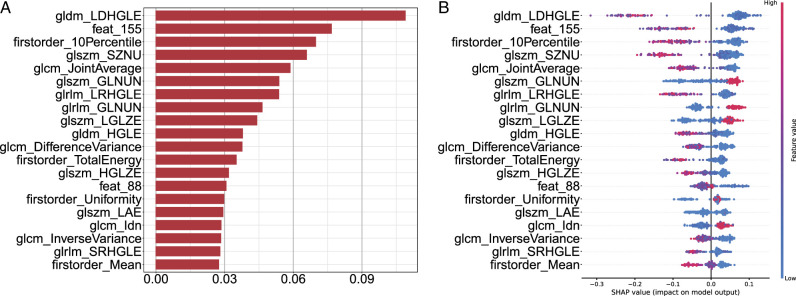
Ranking of SHAP values for the explanation of the proposed model making-decision in training set. Bar-plots (A) and bee-plots (B) display the top-20 drivers’ features SHAP values in ESWL model (success vs. failure).

In order to evaluate the impact of input features on the model predictions, we computed SHAP values for each feature across every sample and presented the resulting SHAP values for all pivotal features through a bar chart. The findings (Fig. 5) suggest that whether morphological features, first-order statistical features, or texture features, all input features contributed to the model’s decision. Particularly noteworthy are specific features, such as gldm Large Dependence High Gray Level Emphasis (gldm_LDHGLE), feat_155, and firstorder 10 Percentile, which exhibited dominant effects in the predictive model concerning the therapeutic outcomes of single-session ESWL for ureteral stones (success vs. failure).

## Discussion

Ureteric calculi, a prevalent urological ailment, profoundly influences the well-being of afflicted individuals. ESWL emerges as a frequently utilized therapeutic modality for ureteric stones, presenting merits such as convenience, minimal invasiveness, diminished patient discomfort, and expeditious postoperative recuperation. Nonetheless, the applicability of ESWL is circumscribed by diverse factors, and incomplete fragmentation of stones may necessitate the repetition of ESWL sessions or resorting to more invasive surgical procedures, thereby giving rise to heightened morbidity, elevated complication rates, and augmented treatment expenditures^11^. Consequently, prognosticating the therapeutic outcomes of single-session ESWL for ureteric calculi (success vs. failure) antecedent to the intervention assumes paramount significance.

In recent times, AI has ascended in prominence both within and beyond the scientific community^12^. CT imaging diagnostic models and algorithms grounded in AI technology have been devised and deployed across diverse medical realms^13–16^, with research indicating their superiority over human experts in intricate medical image or data analysis^17–19^. Presently, some studies have achieved predictions of the therapeutic outcomes of ESWL for ureteral stones, often relying on radiology experts manually measuring imaging indices and signs such as stone size (transverse and longitudinal maximum distance), SSD (skin-to-stone distance), and others^7,20^. Even studies not directly measuring these indices and signs still necessitate manual annotation of the ROI by radiology experts for subsequent texture analysis (TA)^21^. Given the limitations of time-consuming and labor-intensive manual annotation, susceptibility to label noise, and significant subjective influence, our study trained an automated segmentation model to identify and automatically segment the ROI, thereby improving segmentation efficiency. Moreover, we computed the Dice coefficient between automatically segmented and manually annotated ROIs to quantify the overlap between predicted and true segmentations, validating the model’s performance. The average Dice coefficient for this task was 0.88±0.08, indicating a high degree of overlap between the segmented results generated by the model and the true segmentations. This demonstrates the high accuracy and consistency of our automated segmentation model, making it more suitable for large-scale datasets.

Moreover, the majority of extant investigations have not substantiated the explicability of their models^22,23^. The enigmatic quality of these models, devoid of explicability, hinders clinicians from comprehending or rectifying model decisions based on clinical expertise, thereby imposing constraints on clinical applications. To mitigate these constraints, we have employed SHAP values to systematically scrutinize the correlations between diverse indicators and model decisions. In contrast to alternative explicability methodologies such as class activation maps (CAM), SHAP values enumerate the influence of each feature for each case across the entire dataset, facilitating a more profound comprehension of model decisions and even the rationales behind misclassifications. The hive plot, a visual manifestation of SHAP values, is commonly utilized to illustrate the distribution of SHAP values for multiple samples. In Figure 5, the hive plot illustrates that the Gray Level Dependence Matrix (GLDM) feature gldm_LDHGLE exerts the most significant influence on the ESWL efficacy prediction model decision. LDHGLE, an advanced statistical feature employed to assess the spatial dependence and correlation between pixels with elevated gray levels in medical images^24^, mirrors the textural attributes of tissues. A heightened LDHGLE signifies a greater adjacency of voxels with elevated gray level values. In our inquiry, LDHGLE wielded the most pronounced impact on the model decision. This can be elucidated by the divergence in textural characteristics between stones and surrounding tissues, where LDHGLE can quantitatively delineate the textural features of the stone surface, imparting information about the composition and density of the stone. Consequently, we hypothesize that LDHGLE could serve as a prospective indicator for evaluating the efficacy of single-session ESWL in treating ureteral stones. Furthermore, the auto-encoder features we designed, such as feat_155, have also exerted a significant influence on the model’s decisions. These are abstract mathematical features in 3D that do not yet have physical meaning. This feature may represent a certain pixel in the image and could potentially be correlated with the chemical composition of the stones. This abstract correlation warrants further exploration in the future to uncover more unknown value in medical images.

This study utilized a plethora of machine learning algorithms, encompassing SVM, RF, XB, and CB, to fabricate classifiers for prognosticating the therapeutic ramifications of ESWL on ureteral stones. Each algorithm harbors distinctive merits and classification methodologies. SVM is esteemed for its adeptness in managing high-dimensional data and intricate, nonlinear correlations, employing kernel functions to demarcate decision boundaries amidst intricate or inseparable categories. RF embodies an ensemble learning paradigm, fashioning numerous decision trees and amalgamating their prognostications to refine precision and curtail overfitting. It manifests resilience to noisy datasets and demonstrates competence in handling voluminous datasets replete with dimensions. XB, a potent gradient boosting algorithm, consecutively constructs a cascade of feeble learners and amalgamates their prognostications to engender a potent learner, achieving preeminence in myriad machine learning competitions. CB, bespoke for the efficient handling of categorical features, stands as a gradient-boosting algorithm. It autonomously manages absent data and manifests commendable performance under default hyperparameters, necessitating minimal calibration. Amidst these algorithms, CB distinguishes itself for its superior treatment of categorical features, autonomously processing categorical variables and abating the necessity for preprocessing and encoding. Furthermore, CB integrates innovative methodologies such as ordered boosting and oblivious trees, augmenting its efficacy and robustness. Our findings additionally evince that CB surpasses the remaining three algorithms for our specific task and dataset. It attained an AUROC of 0.90 on the internal validation set and 0.83 on the external testing set, evincing robust predictive prowess.

Presently, deep learning paradigms have attained a degree of triumph in prognosticating the efficacy of ESWL for urolithiasis. Seckiner *et al*.^7^ adeptly foretold the outcome of ESWL concerning renal calculi through the application of artificial neural networks (ANN). Choo *et al*.^25^ employed methodologies rooted in machine learning to foresee the effectiveness of ESWL for ureteral stones. However, the resilience of these models remains unverified. If prognostic models are to find application in clinical realms, due consideration must be accorded to their safety and usability. In contradistinction to myriad other investigations within this domain lacking the requisite verification of robustness, our inquiry accentuates the enhancement of model robustness. We deployed a fivefold cross-validation methodology to warrant that our findings bear greater relevance to real-world scenarios. The utilization of fivefold cross-validation is a widely accepted practice in the evaluation of machine learning models, proffering distinct advantages in mitigating the risk of overfitting, diminishing randomness, and augmenting data utilization. In contradistinction to models bereft of robustness verification, our investigation contemplated the significance of model robustness in pragmatic applications, incorporating the fivefold cross-validation method to elevate the model’s robustness. Furthermore, in comparison to studies confining the application of fivefold cross-validation solely to the training queue, neglecting the validation of the external queue and thereby failing to demonstrate the model’s generalization prowess, our inquiry applied cross-validation to external testing tasks.

### Limitations

This investigation is not devoid of its limitations, necessitating refinement. Initially, given its multicenter nature, it grapples with the intricacies of data heterogeneity, posing challenges in the model’s training. Our framework envisions a more adept performance when applied to an extensive and standardized surgical dataset. Secondly, the external cohort, comprising a mere 67 participants, is notably confined in scope. A broader and more diversified external cohort would assuredly bolster the robustness of the study findings. Thirdly, this investigation primarily relies on CT images as the cornerstone for formulating the predictive model. The inclusion of additional clinical information or parameters holds the potential to enrich the development of a more sophisticated and comprehensive predictive model. Therefore, in future research, we intend to explore the integration of clinical information and image features to develop a more comprehensive and applicable predictive model that better serves clinical practice.

## Conclusions

This investigation harnessed the power of machine learning to cultivate an AI prognostication model rooted in CT images. This model introduces a pioneering quantitative methodology for the antecedent prognostication of the effectiveness of ESWL in managing ureteral stones, distinguishing between success and failure. To augment the lucidity of the prognostication model, we additionally employed SHAP values to meticulously scrutinize the quantitative ramifications of CT textural attributes on the model’s determinations. Our model substantiated that the CT textural attributes assume a pivotal role in prognosticating the antecedent efficacy of ESWL for ureteral stones. Albeit further corroboration is imperative for these findings, we posit that the outcomes of this inquiry harbor noteworthy clinical applicability, proffering bespoke treatment stratagems for patients contending with ureteral stones antecedent to ESWL.

## Ethical approval

This study has received ethical approval from the Ethics Committee 2023-KYLL-57, the third affiliated hospital of Shenzhen University, located in Shenzhen, China, on 13 November 2023.

## Consent

We have obtained written informed consent from the patient for publication, including any relevant images. Copies of the written consent are available for the journal’s editor to review upon request.

## Source of funding

This study has received funding from the Shenzhen Medical Research Fund (A2301007, A2301008) and the Guangdong Basic and Applied Basic Research Foundation (2019A1515110038).

## Author contribution

H.Y.: methodology, investigation, writing – original draft preparation; X.W.: methodology, visualization, writing – reviewing and editing; W.L.: methodology, investigation, writing – reviewing and editing; Z.Y. and T.W.: data curation, software, and validation; W.Y., B.Y., and B.W.: data curation and software; K.W.: conceptualization, funding acquisition, writing – reviewing and editing; H.Z.: conceptualization, funding acquisition, writing – reviewing and editing; H.L.: resources, supervision, writing – reviewing and editing.

## Conflicts of interest disclosure

The authors of this manuscript declare no relationships with any companies, whose products or services may be related to the subject matter of the article.

## Research registration unique identifying number (UIN)

This study only analyzed clinical and radiological data and did not intervene in the diagnosis and treatment of patients.

## Guarantor

Kai Wu, Haoyang Zeng, and Hanlin Liu.

## Data availability statement

The code and datasets used in this study are available upon reasonable request from the corresponding author.

## Provenance and peer review

Not commissioned, externally peer-reviewed.
